# Dexamethasone intravitreal implants in the management of tubercular multifocal serpiginoid choroiditis

**DOI:** 10.1186/s12348-016-0101-4

**Published:** 2016-08-30

**Authors:** Alex Fonollosa, Sonia Valsero, Joseba Artaraz, Ioana Ruiz-Arruza

**Affiliations:** 1 Department of Ophthalmology, BioCruces Health Research Institute, Cruces Hospital, University of the Basque Country, Bilbao, Spain; 2 Autoimmune Diseases Research Unit, Department of Internal Medicine, BioCruces Health Research Institute, Cruces Hospital, University of the Basque Country, Bilbao, Spain; 3 Department of Ophthalmology, Cruces University Hospital, Plaza de Cruces s/n, Cruces-Barakaldo, CP 48903 Vizcaya, Spain

**Keywords:** OZURDEX, Dexamethasone intravitreal implant, Uveitis, Multifocal serpiginoid choroiditis, Tuberculous uveitis

## Abstract

**Background:**

Continuous progression of lesions despite an adequate treatment has been described in tubercular multifocal serpiginoid choroiditis. Reported treatments for this paradoxical response include systemic steroids, immunosuppressive drugs, and intravitreal methotrexate. We describe the use of dexamethasone intravitreal implants in a patient presenting with this condition.

**Findings:**

A 46-year-old woman sought medical attention for scotomas in her left eye. Tests suggested multifocal serpiginoid choroiditis associated with latent tuberculosis infection, and hence, she was started on anti-tuberculosis drugs in combination with corticosteroids. Given that lesions progressed despite this treatment, we began treatment with dexamethasone intravitreal implants. After injection of the second implant, we succeeded in inactivating the inflammatory process.

**Conclusions:**

Dexamethasone intravitreal implants may be a suitable alternative to systemic steroids or immunosuppressive therapy in the management of continuous progression of lesions in tubercular multifocal serpiginoid choroiditis.

## Findings

### Introduction

Multifocal serpiginoid choroiditis (MSC) is a rare entity producing chronic recurrent progressive inflammation of the retinal pigment epithelium and choriocapillaris that is associated with latent tuberculosis infection [1]. Treatment consists of anti-tuberculosis antibiotics and corticosteroids. A paradoxical response to treatment has been described in some patients with MSC, consisting of progression of the disease despite appropriate treatment. We describe the case of a patient with this condition who was treated with dexamethasone intravitreal implants to manage this paradoxical response.

### Case report

**Fig. 1:**
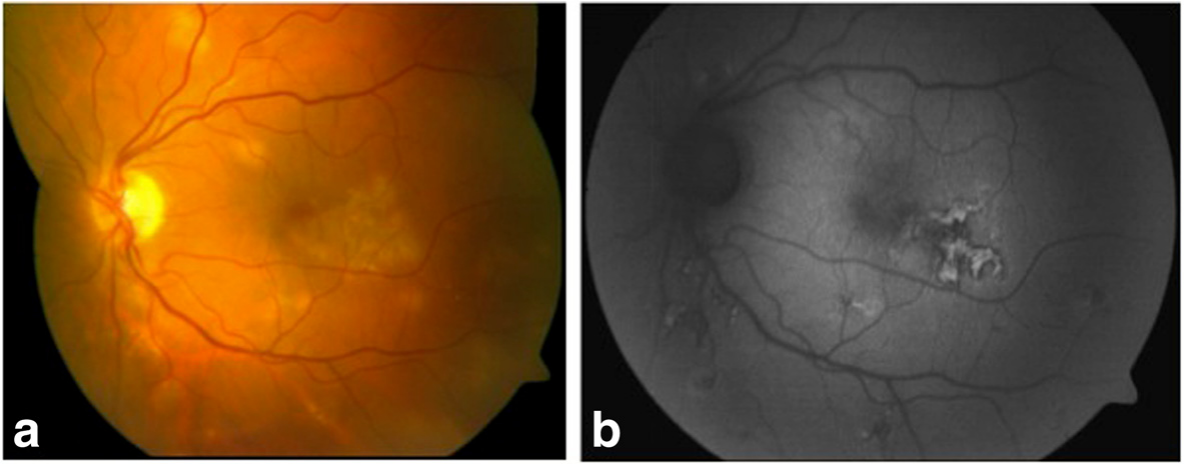
Retinography **(a)** and fundus autofluorescence imaging **(b)** on admission showing multifocal chorioretinitis lesions

A 46-year-old women with myopia (7 dioptres) in both eyes sought medical attention for a 1-month history of paracentral scotomas in her left eye. Her personal history included contact with a patient with tuberculosis 20 years earlier and a tick bite 10 years earlier with no clinical manifestations. Eye examination found a best-corrected visual acuity of 1.0 in both eyes, anterior segment slit lamp biomicroscopy was normal in both eyes, and the intraocular pressure was 19 mmHg in both eyes. Funduscopic examination was normal in the right eye but revealed multifocal serpiginoid lesions in the posterior pole and mid-peripheral retina of the left eye, with some cells in the vitreous body (Fig. 1).

Requests were made for the following: serologies for syphilis, *Borrelia burgdorferi*, hepatitis B and C, and HIV, for which results were normal; quantiFERON (Cellestis, Victoria, Australia) and Mantoux tests, which were positive; and a chest X-ray, which produced no abnormal findings.

**Fig. 2:**
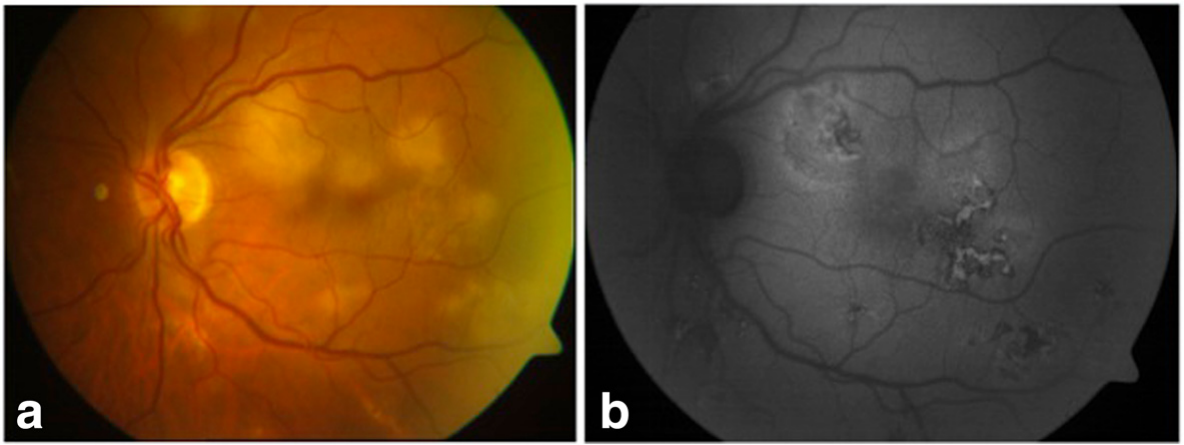
Retinography **(a)** and fundus autofluorescence imaging **(b)** showing the progression of lesions. The patient had initiated anti-tuberculosis treatment and tapered doses of oral prednisone. When she decreased the dose to 10 mg, new lesions started to appear

**Fig. 3:**
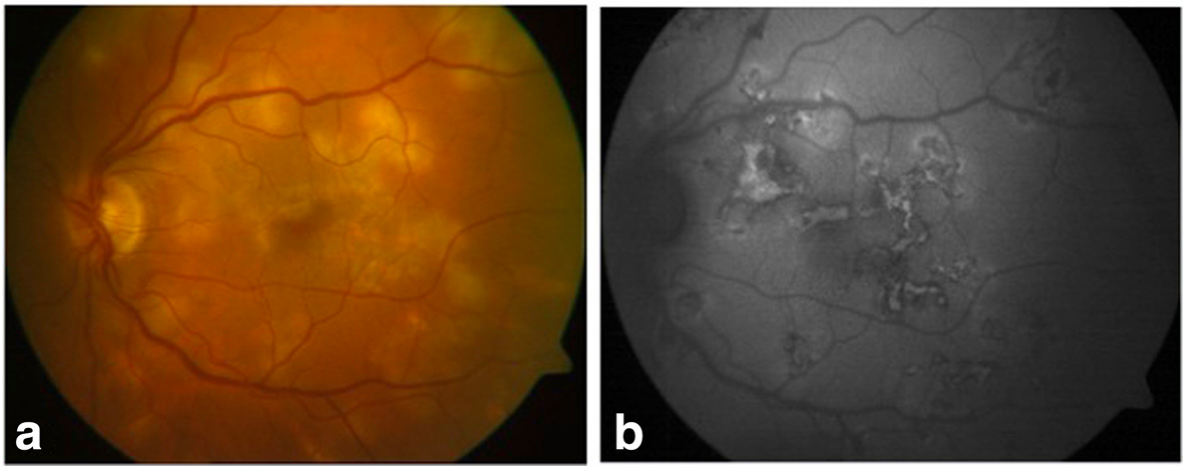
Retinography **(a)** and fundus autofluorescence imaging **(b)** showing new lesions. The patient had initiated a second tapered course of prednisone. At this point, a dexamethasone intravitreal implant was indicated

**Fig. 4:**
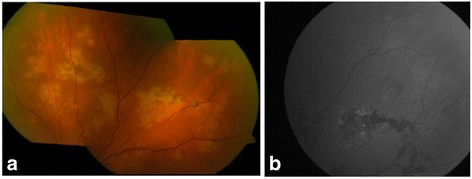
Retinography **(a)** and fundus autofluorescence imaging **(b)** showing the progression of lesions 5 months after the injection of dexamethasone intravitreal implant. A second injection was given

**Fig. 5:**
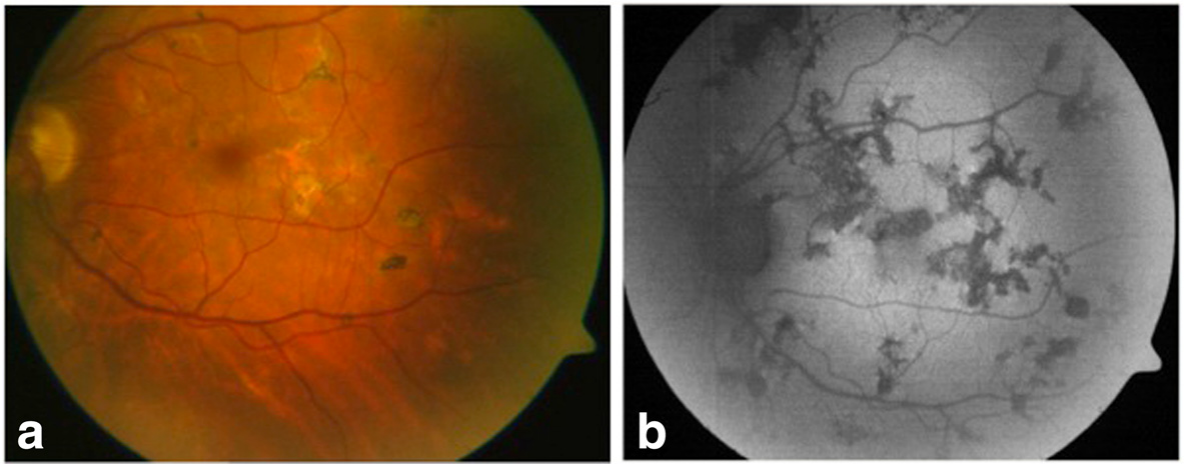
Retinography **(a)** and fundus autofluorescence imaging **(b)** at the last follow-up visit (12 months after the second dexamethasone intravitreal implant) showing healed lesions

We established the diagnosis of MSC and initiated treatment with anti-tuberculosis drugs: ethambutol, isoniazid, pyrazinamide, and rifampicin for 2 months, together with a tapered dose of oral prednisone (with a starting dose of 30 mg). Initially, lesions stabilised but when we decreased the dose of corticosteroids to 10 mg (in the third week of treatment), new lesions started to appear (Fig. 2). We attempted to control the inflammation with three intravenous pulses of methylprednisolone (250 mg) and initiated a second tapered course of corticosteroids, but once again, after initial stabilisation, the lesions recurred (Fig. 3). At that point, 2 months after having initiated the antibiotic treatment, we decided to treat the patient using a dexamethasone intravitreal implant (OZURDEX; Allergan, Inc., Irvine, CA). This stopped the progression of the lesions, and the antibiotic treatment with rifampicin and isoniazid was continued (completing 9 months of treatment). The intraocular pressure was 19 mmHg in all subsequent checkups. Five months later, we observed new lesions, and hence, a second implant was used (Fig. 4). After this second injection, lesions became inactive and no new inflammatory foci have been observed, the intraocular pressure remaining at 19 mmHg at the most recent follow-up, 12 months after this second treatment (Fig. 5). Visual acuity at this point was 1.0 in both eyes.

### Discussion

MSC is a type of posterior uveitis characterised by recurrent and chronic inflammation of the retinal pigment epithelium and choriocapillaris, associated with latent tuberculosis infection [1]. The usual treatment consists of administering anti-tuberculosis antibiotics together with oral corticosteroids. It has been reported, however, that lesions progress in some patients, despite appropriate treatment for tuberculosis. This paradoxical response has been attributed to an elevated immune response to the release of tubercular antigens; antibiotics may trigger the release of these antigens as the mycobacteria are lysed [2]. A remarkable point is that rifampicin, a commonly used anti-tuberculosis drug, decreases bioavailability of corticosteroids; hence, this may contribute to the progression of inflammation. In fact, the dose of corticosteroids needs to be doubled when patients are treated with rifampicin [3].

Gupta et al. [4] described the progression of the disease in 12 out of 84 patients (14 %) with MSC. The continued progression was managed by increasing the dosage of oral prednisolone in nine patients (two of them receiving also intravenous metilprednisolone) while in four patients, immunosuppressive agents (cyclophosphamide or azathioprine) were given to control the condition. In the other three patients, the dosage of oral corticosteroid was not increased further. Basu et al. [5] described the progression of the disease in 26 out of 106 patients (24.5 %) with different types of presumed tuberculous uveitis; 24 patients had MSC, and of these, seven showed progression of the disease; all of these patients were effectively managed by increasing the dose of corticosteroids and continued antibiotics. Julian et al. [6] described the use of intravitreal methotrexate in patients with MSC with active disease despite treatment with systemic antibiotics. In their series of three eyes from two patients, choroidal lesions healed within the first month after the injection. Since dexamethasone intravitreal implants have been shown to be very effective in managing intraocular inflammation both in non-infectious [7] and infectious uveitis [8], and a local approach has the advantage of reducing the risk of activating tubercular systemic disease, we considered this treatment a suitable option for our patient.

The treatment was effective, though two injections were required. For 5 months after the first injection, we observed healing of the lesions and no new ones appeared. After this period, when the effect of the implant would be expected to have worn off, recurrence was observed, and this was effectively managed with a second dexamethasone intravitreal implant. Though a paradoxical response is more frequent early after initiating antibiotic treatment, cases developing after as long as 5 months have been described [3]. The second flare-up we observed falls into this category of late recurrences.

We believe that in such cases of MSC with a paradoxical recurrence of inflammation, use of a dexamethasone intravitreal implant, to our knowledge, not previously described for this condition, may be a suitable alternative to systemic immunosuppressive therapy. Local management of the condition would avoid inducing severe immunosuppression in patients with a latent tuberculosis infection and hence, the associated risk of developing active tuberculosis. On the other hand, serpiginoid inflammatory lesions in the posterior segment of the eye (serpiginous-like choroiditis) may be caused by infectious aetiologies other than tuberculosis so they should be ruled out before giving a dexamethasone intravitreal implant to treat this condition [9].

## Abbreviations

MSC: Multifocal serpiginoid choroiditis
HIV: Human immunodeficiency virus
